# Comparison of specific endophytic bacterial communities in different developmental stages of *Passiflora incarnata* using culture‐dependent and culture‐independent analysis

**DOI:** 10.1002/mbo3.896

**Published:** 2019-08-27

**Authors:** Marcela C. Goulart, Luis G. Cueva‐Yesquén, Kelly J. Hidalgo Martinez, Derlene Attili‐Angelis, Fabiana Fantinatti‐Garboggini

**Affiliations:** ^1^ Graduate Program in Genetics and Molecular Biology, Institute of Biology University of Campinas (UNICAMP) Campinas Brazil; ^2^ Division of Microbial Resources (DRM), Research Center for Agricultural, Biological and Chemical (CPQBA) University of Campinas (UNICAMP) Paulínia Brazil

**Keywords:** 16S rRNA gene sequencing, diversity, endophytic microbiome, microbial ecology, plant development

## Abstract

Plants and endophytic microorganisms have coevolved unique relationships over many generations. Plants show a specific physiological status in each developmental stage, which may determine the occurrence and dominance of specific endophytic populations with a predetermined ecological role. This study aimed to compare and determine the structure and composition of cultivable and uncultivable bacterial endophytic communities in vegetative and reproductive stages (RS) of *Passiflora incarnata*. To that end, the endophytic communities were assessed by plating and Illumina‐based 16S rRNA gene amplicon sequencing. Two hundred and four cultivable bacterial strains were successfully isolated. From the plant’s RS, the isolated strains were identified mainly as belonging to the genera *Sphingomonas*, *Curtobacterium*, and *Methylobacterium*, whereas *Bacillus* was the dominant genus isolated from the vegetative stage (VS). From a total of 133,399 sequences obtained from Illumina‐based sequencing, a subset of 25,092 was classified in operational taxonomy units (OTUs). Four hundred and sixteen OTUs were obtained from the VS and 66 from the RS. In the VS, the most abundant families were Pseudoalteromonadaceae and Alicyclobacillaceae, while in the RS, Enterobacteriaceae and Bacillaceae were the most abundant families. The exclusive abundance of specific bacterial populations for each developmental stage suggests that plants may modulate bacterial endophytic community structure in response to different physiological statuses occurring at the different plant developmental stages.

## Graphical abstract

This paper deals with the comparison of endophytic bacterial diversity between two stages of development of *Passiflora incarnata*. The results demonstrate the existence of specific communities during the reproductive stage and the vegetative stage of the plant.

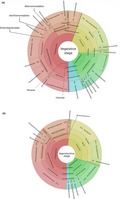

## 1 INTRODUCTION

Microbial endophytes are part of the plant micro‐ecosystem, where they live internally without causing any damage or apparent symptom of a disease. These endophytes are ubiquitously associated with almost all plants (Nair & Padmavathy, 2014; Sharma, Kansal, & Singh, 2018). Endophytic bacteria colonize plant’s intracellular or intercellular spaces and may originate from the phyllosphere, rhizosphere, or even seeds, existing in both free‐living or endophytic states (Farrar, Bryant, & Cope‐Selby, 2014). For the establishment of this plant–microbe interaction, plants constituted a complex ecosystem where they can provide necessary nutrients for microbial colonization. In return, endophytes perform diverse beneficial functions for the host‐plant. They may directly affect the plant’s development by making essential nutrients more available and modulating levels of phytohormones (Ryan, Germaine, Franks, Ryan, & Dowling, 2008; Tsavkelova, Klimova, Cherdyntseva, & Netrusov, 2006), or, as an indirect effect, through the synthesis of biomolecules, they may provide protection against abiotic and biotic stresses (Guo, Wang, Sun, & Tang, 2008; Strobel & Daisy, 2003). Thus, the plant may select its internal microbial population toward a specific ecological role to be played in this ecosystem (Hardoim et al., 2015; da Silva, Armas, Soares, & Ogliari, 2016). The plant‐related factors known to determine the structure and composition of endophytic communities are the plant genotype, developmental stage, and crop environmental conditions (İnceoğlu, Salles, Overbeek, & Elsas, 2010; Van Overbeek & Van Elsas, 2008; Ren, Zhang, Lin, Zhu, & Jia, 2015; Yu, Yang, Wang, Li, & Yuan, 2015). Considering phenological aspects of plants, endophytic communities may also respond to seasonal conditions, as their hosts go through different developmental stages with each season.

Several methods have been progressively developed for analyzing the structure and composition of the host‐associated microbial communities. Culture‐dependent methods are suitable for functional studies of native species but are limited as it is estimated to recover <1% of the total bacterial diversity. It is known that conventional microbiological techniques select for specific groups that are able to grow under preestablished isolation conditions (Stewart, 2012; Vartoukian, Palmer, & Wade, 2010). In contrast, culture‐independent methods may detect the occurrence of uncultivable, slow‐growing, or less abundant bacteria. These methods, generally based on 16S rRNA gene sequencing (Tringe & Hugenholtz, 2008), can be high throughput to assess the composition of bacterial communities in soil, water, air, or any environmental sample (An, Sin, & DuBow, 2015; Doherty et al., 2017; Janssen, 2006; Shokralla, Spall, Gibson, & Hajibabaei, 2012).

Passionflower is a tropical plant of the family Passifloraceae, mainly distributed throughout North and South America (Dhawan, Dhawan, & Sharma, 2004). In Brazil, the species grows into the vegetative stage (VS) from December to January, and the reproductive stage (RS) is (flowering and fruiting) from April to November (Fuentes, Lemes, & Rodríguez, 2000). It grows preferentially in well‐drained soil, forming a climbing stem, three‐lobed leaves, ovoid or globose fruits, and, due to the exotic appearance of its flower, it is recognized as the symbol for the “Passion of the Christ” (Miroddi, Calapai, Navarra, Minciullo, & Gangemi, 2013; Patel, Verma, & Gauthaman, 2009). *P.  incarnata* has been widely used in traditional herbal medicine for treating anxiety, nervousness, constipation, dyspepsia, and insomnia. Nowadays, it is officially included in the national pharmacopeias from France, Germany, and Switzerland, also being monographed in the British Herbal Pharmacopoeia and the British Herbal Compendium (Dhawan et al., 2004). Although its therapeutic aspects have been widely reported, only one study on *P.  incarnata* fungal endophytes was performed (Seetharaman et al., 2017). The present study is the first one to evaluate the bacterial endophytic diversity associated with this medicinal plant.

The analysis of the structure of plant‐associated bacterial communities in their different stages of development may establish a correlation between the occurrence of specific bacterial populations and physiological changes throughout the plant’s development. These plant‐related conditions may play a critical role in the modulation of the endophytic communities. This study aimed at determining and comparing the structure and composition of cultivable and uncultivable bacterial endophytic communities to be found in the vegetative and RS of *P.  incarnata*.

## 2 MATERIAL AND METHODS

### 2.1 Sample collection and surface sterilization

*P.  incarnata* L. cv. CF 01 leaves were collected in April 2015 and January 2016 from the Centroflora Group agricultural fields located at Botucatu, state of São Paulo, Brazil (22º56′23.4″S 48º34′11.6″W). This area is mountainous, with altitudes ranging between 700 and 900 m, displaying a humid subtropical climate with a mean temperature of 22°C in January and 22.6°C in April. Regarding its phenology, *P.  incarnata* is typically in the VS in January, while in April it develops into its RS. Thirty healthy plants were randomly sampled in April 2015 for the RS, and these plants were flagged for the next sampling. In January 2016, sampling from the previously flagged plants was carried out, but these were in the VS. Sterilized gloves and scalpels were used to collect the whole leaves; the blades were changed between each collection. The samples were placed in sterilized polythene bags, transported to the laboratory on ice, and stored at 4°C until they were ready to be processed up to 72 hr afterward. The leaves were detached with a sterilized scalpel, washed with purified distilled water, and left 10–15 min to drain. Surface sterilization was performed on whole leaves according to Azevedo, Maccheroni, Pereira, and Araújo (2000), with modifications. Leaf tissues were treated with 100% ethanol for 3 min, followed by 2% sodium hypochlorite for 2 min, and 70% ethanol for 3 min. The disinfected leaves were washed three times with sterilized distilled water, and the last washing was inoculated on nutrient agar plates to validate the effectiveness of the surface sterilization procedure. Control agar plates incubated at 28 ± 2ºC were inspected for 48 hr to check the occurrence of any bacterial growth.

### 2.2 Culture‐dependent diversity

#### 2.2.1 Isolation of endophytic bacteria

Surface‐sterilized leaves (five per plant) were ground with sterile mortars and pestles in 5 ml phosphate buffer saline (137 mM NaCl, 2.7 mM KCl, 10 mM Na_2_HPO_4_ and 1.8 mM KH_2_PO_4_, distilled water 1,000 ml, pH 7.4) to provide a mixed sample for the isolation of bacterial strains. From the resulting suspension, a series of 10‐fold dilutions down to 10^−4^ were prepared. Aliquots (100 µl) of each dilution were spread in triplicates on M9 minimal medium, Gause’s synthetic agar (Zhao, Xu, & Jiang, 2012), Chitin medium (Lingappa & Lockwood, 1961), Tap Water Yeast Extract agar (El‐Shatoury, 2013), Humic acid‐vitamin (HV) agar (Hayakawa & Nonomura, 1987), Glycerol–asparagine agar (Pridham & Lyons, 1961) and 869 medium (Eevers et al., 2015). All media were supplemented with benomyl (50 µg/ml) and cycloheximide (50 µg/ml). Plates were incubated at 28 ± 2ºC for up to 30 days. Endophytic bacterial strains, isolated from surface‐sterilized leaves, were selected based on colony morphologies, purified, and preserved at −80°C. Margalef index (D_MG_) was calculated to determine the species richness of bacterial populations isolated on each culture medium.

#### 2.2.2 DNA extraction and 16S rRNA gene sequencing

The genomic DNA of the endophytic bacterial strains was extracted using the methods described by Van Soolingen, Haas, Hermans, Groenen, and Embden (1993), with modifications. The 16S rRNA gene was amplified using universal bacterial 16S ribosomal gene primers 10F (5′‐AGTTTGATCCTGGCTCAG‐3′) and 1525R (5′‐AGTTTGATCCTGGCTCAG‐3′) (Lane, 1991) targeting the V1–V9 region. The 25 µl PCR reaction mixture contained 10 ng of DNA, 0.5 µl of dNTP mix (10 mM; Applied Biosystems), 2.5 µl of 10X PCR Buffer with 15 mM MgCl_2 _(Applied Biosystems), 0.5 µM of each primer, one unit of Taq DNA polymerase (Applied Biosystems). The PCR conditions consisted of initial denaturation at 95ºC for 2 min, followed by 35 cycles of 94ºC for 1 min, 60ºC for 1 min, 72ºC for 3 min, and a final extension at 72ºC for 5 min. Agarose gel electrophoresis separated the PCR products, purified using GFX^TM^ PCR DNA and Gel Band Purification kit (GE Healthcare Life Sciences, Germany), and sequenced on an ABI3500XL Series (Applied Biosystems) sequencer. The primers above mentioned were used to assembly the 16S rRNA gene sequence, and the 1100R (5′‐AGGGTTGGGGTGGTTG–3′) was used as an internal sequencing primer. For taxonomic assignment of bacterial strains, the 16S rRNA gene sequences were compared with the EZBiocloud 16S Database using the “Identify” service (Yoon et al., 2017), and species assignment was based on closest hits (Kim & Chun, 2014).

### 2.3 Culture‐independent diversity

#### 2.3.1 DNA extraction and Illumina‐based sequencing

The leaf samples obtained from the same plants used for the isolation of cultivable bacterial communities were sterilized using the same conditions described previously. Sterilization was confirmed by running a PCR with the same primers previously used on the last washing, and if no DNA was detected after the amplification, the sterilization was considered successful. The sterilized leaf tissues were homogenized in sterile mortars and pestles with PBS solution. The total genomic DNA was extracted using a PowerMax Soil DNA Extraction kit (Mo Bio Laboratories, Carlsbad, CA), following the manufacturer’s instructions. DNA from the 30 replicates collected in the VS and from the 30 replicates collected in the RS were pooled to create a single DNA sample for the VS and a single DNA sample for the RS. These two DNA samples were used as templates for the culture‐independent approach. The 16S rRNA gene V3‐V4 hypervariable region was amplified with primers 799F (5′‐AACMGGATTAGATACCCKG‐3′) and 1492R (5′‐GGTTACCTTGTTACGA CTT‐3′) (Chelius & Triplett, 2001) with a barcode on the forward primer. The PCR reaction was performed in 30 cycles (five cycles used on PCR products) using the HotStarTaq Plus Master Mix Kit (Qiagen) under the following conditions: 94°C for 3 min, followed by 28 cycles of 94°C for 30 s, 53°C for 40 s and 72°C for 1 min, and a final elongation step at 72°C for 5 min. Amplification products were checked in 2% agarose gel to determine the success of amplification and the relative intensity of bands. Amplicon sequencing was performed on the Illumina MiSeq platforms at Mr. DNA Molecular Research (Texas).

#### 2.3.2 Processing of sequencing data

Raw sequence data were checked with sequence quality filters in FastQC software (Andrews, 2012). Sequences of lengths < 150 bp were removed, and the adapter, barcodes, and primers were trimmed using Trimmomatic software (version 0.36) (Bolger, Lohse, & Usadel, 2014). The sequencing data were processed using Quantitative Insights into Microbial Ecology (QIIME) software version 1.9.1 (Caporaso et al., 2010). All sequences that passed quality controls were clustered in operational taxonomic units (OTUs) at 97% genetic identity using an open reference approach (UCLUST algorithm) (Edgar, 2010). A representative sequence for each OTU was classified using Ribosomal Database Project classifier (Wang, Garrity, Tiedje, & Cole, 2007) and PyNast aligner (Caporaso et al., 2009) against the SILVA database (128 release) for taxonomy assignment (Quast et al., 2012). The chimeras were checked and filtered out by UCHIME (Edgar, Haas, Clemente, Quince, & Knight, 2011). OTUs assigned to chloroplasts or of mitochondrial origin were excluded. Only OTUs of bacterial origin were considered for further analysis.

Rarefaction curve, alpha‐diversity indices (Shannon‐Wiener Index, Simpson’s evenness Index) and richness estimators (Abundance‐based Coverage Estimator and Chao1) were calculated using the QIIME pipeline. The index estimator Chao was used to estimating the richness of the bacteria. The Shannon diversity and Simpson index were used to estimate the biodiversity of the bacterial communities. In order to calculate the diversity indices, each sample was rarified to an average sequences’ depth, due to the variation in number obtained per sample (de Cárcer, Denman, McSweeney, & Morrison, 2011). In this study, the OTU table was rarefied to 404 sequences, corresponding to the sample with the lowest number of sequences (RS). We normalized this table of good reads by dividing the reads per OTU in a sample by the sum of good reads in that sample, resulting in a table of relative abundances (frequencies). All diversity indices and richness estimators were calculated 10 times. Unassigned sequences were excluded from the determination of contributions of taxonomic groups in each bacterial community. The structure of bacterial endophytic communities was visualized in Krona graphs, plotted using the Krona web interface software (Ondov, Bergman, & Phillippy, 2011).

### 2.4 Statistical analysis

In order to search for biologically meaningful differences in the taxonomic distribution between endophytic bacterial communities in VS and RS, the two‐way Fisher’s exact test with a Storey False Discovery Rate multiple test correction analysis (adjusted *q*‐value < 0.05 and ratio of proportions effect size < 2.00) was carried out using the graphical software package Statistical Analysis of Taxonomic and Functional Profiles (STAMP) (Parks & Beiko, 2010). The normalized OTUs table format was adjusted to generate a heat map with hierarchical cluster based on Bray–Curtis distance using the clustering function hclust2 at R version 3.4.2.

## 3 RESULTS

### 3.1 Culture‐dependent diversity analysis

In total, 204 pure cultures showing different colony morphologies were obtained; 146 were retrieved from the RS and 58 from the VS. No colonies emerged from the final washing of the sterilization procedure, an assurance that the surface sterilization procedure was effective. All isolates were identified based on 16S rRNA gene sequencing and alignment. The sequence data for these isolates have been submitted to the GenBank database under accession numbers from MG778707 to MG778907. Most of the sequences showed > 99% similarity to the reference strains of EzBiocloud database; only 10.3% showed a similarity between 97% and 99%. The results revealed a high diversity, distributed in 84 different bacterial taxa (Appendix Table A1). In the RS, Proteobacteria was the most abundant phylum, comprising 68.5% of total isolates. Alphaproteobacteria was represented mainly by Sphingomonadaceae (32.9%) and Methylobacteriaceae (13.7%), followed by Rhodobacteraceae (4.6%), Bradyrhizobiaceae (1.4%), and one strain of Caulobacteraceae. The class Gammaproteobacteria included Moraxellaceae (7.6%), Pseudomonadaceae and Enterobacteriaceae (2.8%), and Xanthomonadaceae (1.4%). Actinobacteria were the second most abundant phylum (25.9% of total isolates) dominated by Microbacteriaceae (21%), Streptomycetaceae (2.8%), Micrococcaceae (1.4%), and one isolate of the genus *Mycobacterium*. The phylum Firmicutes, mainly represented by bacteria belonging to the genus *Bacillus*, constituted only 4.9% of the total isolates. One isolate of genus *Chryseobacterium* represented the Bacteroidetes. In the VS, Firmicutes was the dominant phylum (76%), uniquely represented by the family Bacillaceae (74%). Proteobacteria was the second most present phylum (21%), comprising Pseudomonadaceae (9%), Erwiniaceae (7%), followed by Enterobacteriaceae (4%) and Xanthomonadaceae (2%). Actinobacteria were represented by the genus *Rhodococcus* (3%).

**Figure 1:**
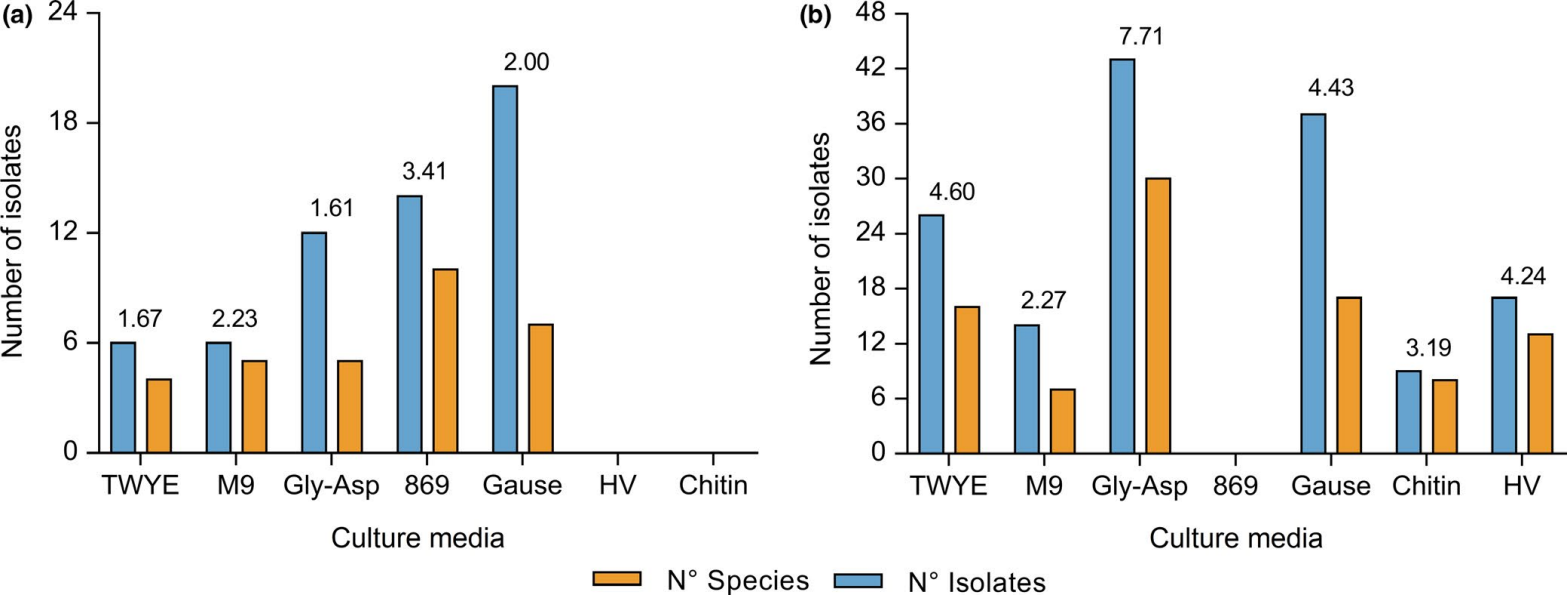
Graphical representation of culturable community recovered in each culture medium. Number of species and isolates from (a) vegetative stage and (b) reproductive stage of *Passiflora* *incarnata*. The values shown above of each pair bars are the richness index of Margalef (D_MG_)

Seven different culture media were used to recover a representative cultivable diversity from the composition of the bacterial community associated with *P.  incarnata*. All agar media were suitable for the isolation of endophytic bacteria. No bacteria were isolated on Chitin and HV media when processing VS samples. Based on D_MG_, the 869 medium recovered the highest species richness (D_MG_ = 3.41) in the VS. By contrast, no bacteria were isolated on the 869 medium from RS samples, and the highest species richness (D_MG_ = 7.71) was obtained from the Glycerol‐Asparagine medium (Figure 1).

### 3.2 Culture‐independent diversity analysis

#### 3.2.1 Bacterial diversity, species richness, and taxonomic distribution

A total of 133,399 sequences (111,335 from VS and 22,064 from RS) was recovered after applying all quality filters. As per QIIME analysis, most sequences from both developmental stages (78% in VS and 98% in RS) showed similarity to chloroplast or mitochondrial 16S rRNA gene, even using the primers designed to avoid this bias. Sequence data have been deposited into the NCBI Sequence Read Archive database with the BioProject No. PRJNA430160.

**Figure 2:**
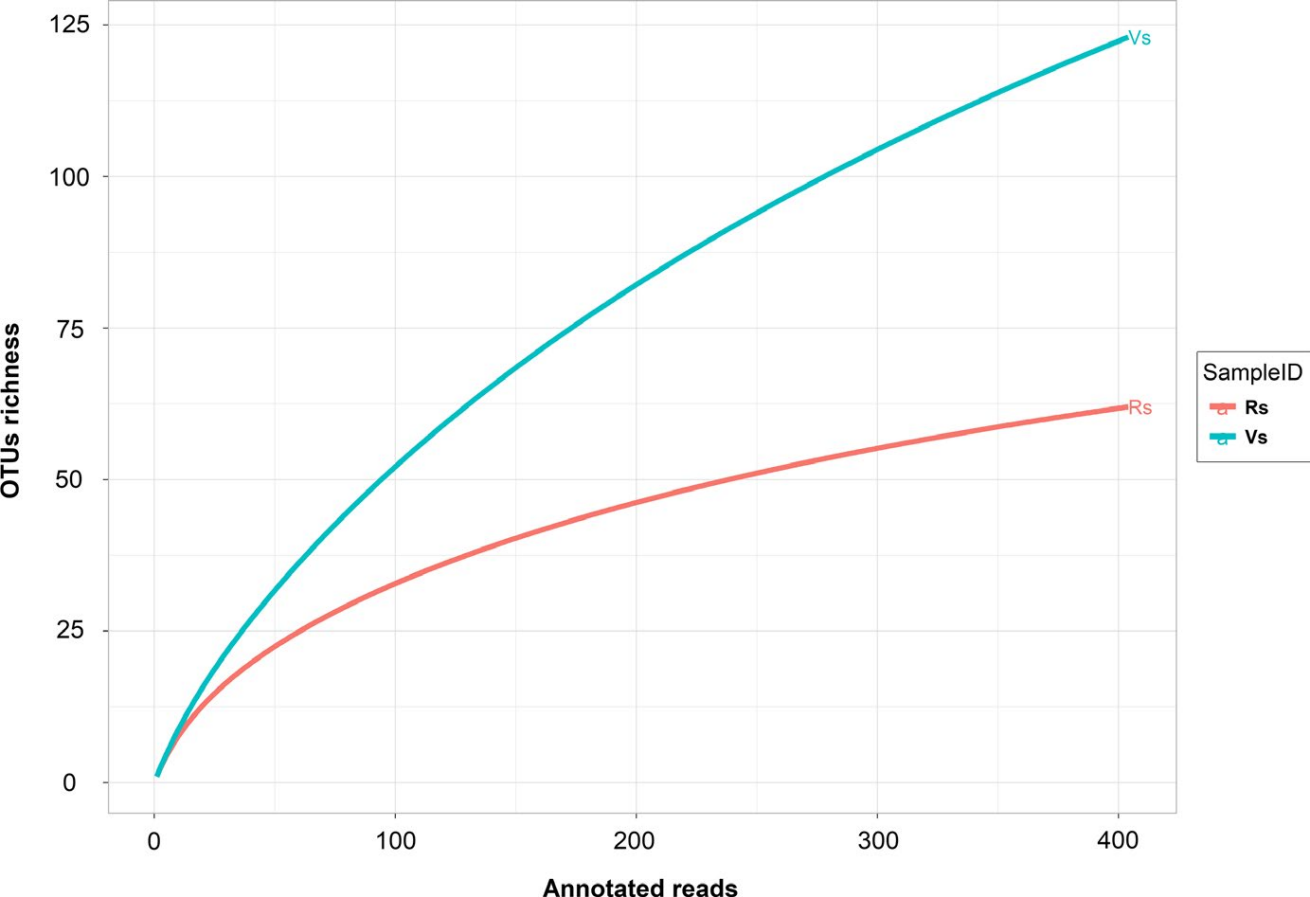
Rarefaction curves of partial sequences of 16S rRNA gene. Rarefaction analysis of 16S rRNA gene sequence data to estimate microbial diversity based on a cutoff <97% sequence identity. Abbreviation: OTUs, operational taxonomy units

After excluding the unassigned sequences, the OTUs table retained 17,526 sequences clustered in 416 OTUs representing the VS and 404 sequences clustered in 66 OTUs representing the RS. The rarefaction curve indicated different diversity profiles between the two samples (Figure 2). The VS rarefaction curve did not reach saturation, suggesting that taxonomic diversity was not fully exploited, while the RS curve tends to reach a plateau, indicating that most of the diversity was recovered. The OTU table was rarefied to 404 sequences per sample before calculation of the alpha‐diversity indices to compare the diversity and species richness in the VS and the RS. Shannon, Simpson, Ace and Chao1 indices (Table 1) confirmed that VS presented the highest species richness.

**Table 1:** Number of OTUs and alpha‐diversity indices of the endophytic bacterial communities associated with *Passiflora incarnata*

Developmental stage	Reads	OTUs^a^	Richness estimators^b^		Diversity indices^b^	
			Ace	Chao1	Shannon	Simpson
Vegetative	17,526	115	192	193	3.992	0.96
Reproductive	404	51	59	58	3.125	0.92

a The operational taxonomic units (OTUs) were defined at a 97% similarity level.

b The coverage percentage, richness estimators (ACE and Chao1) and diversity indices (Shannon and Simpson) were calculated using Quantitative Insights into Microbial Ecology pipeline.

**Figure 3:**
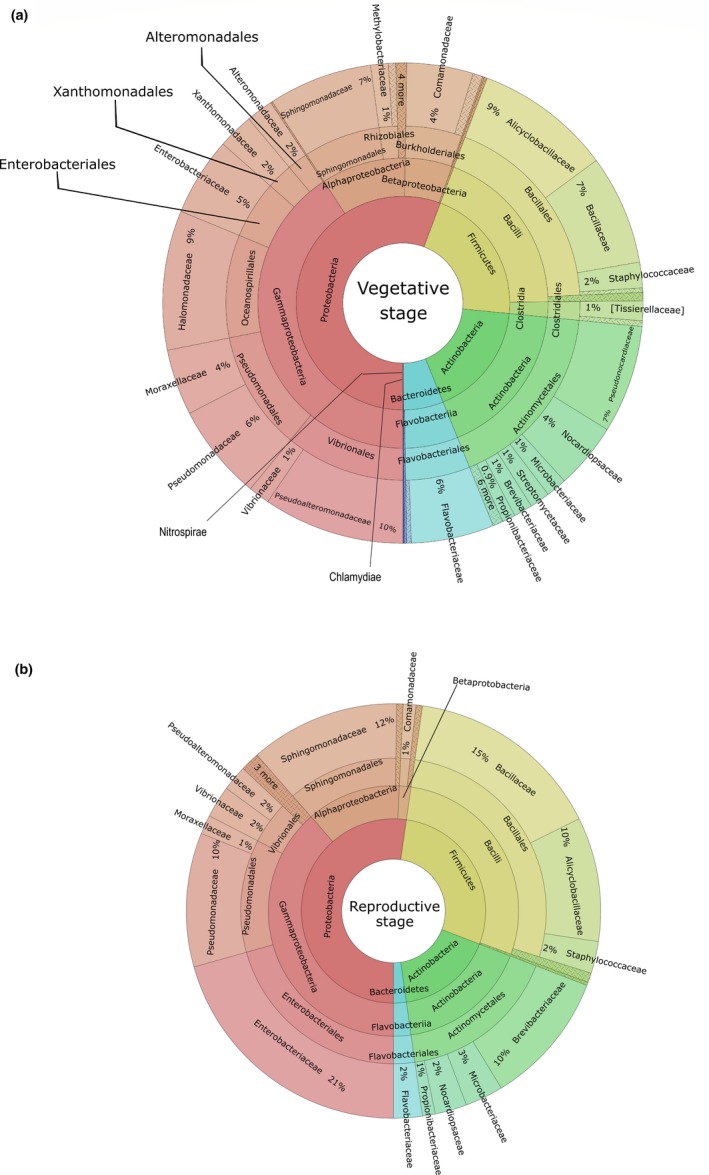
Krona plots on 16S rRNA sequences of the bacterial communities associated with *Passiflora incarnata* leaves. The data represent taxonomic hierarchies of bacterial communities in the (a) vegetative stage and in the (b) reproductive stage in a multilevel diagram

Given that 98.4% of the sequences could be classified at the family level, and 70.1% of sequences could be classified at the genus level, the taxonomic composition was represented at the family level. Taxonomic composition in each developmental stage was plotted into Krona charts (Figure 3).

The sequences from the VS were classified into six different phyla, 11 classes, 24 orders, 54 families and 51 genera; the sequences from RS were classified into four different phyla, seven classes, 15 orders, 23 families, and 18 genera. Most of the identified OTUs in both stages belong to the phylum Proteobacteria (56.2% in the VS and 52.5% in the RS). Other bacterial phyla found in the VS were Firmicutes (20.7%), Actinobacteria (17.1%), Bacteroidetes (5.9%), Nitrospirae (0.08%) and Chlamydiae (0.01%). The other three dominant phyla in the RS were Firmicutes (28.5%), Actinobacteria (16.8%) and Bacteroidetes (2.2%). In the VS, the most abundant families were Pseudoalteromonadaceae, Alicyclobacillaceae, and Bacillaceae, representing 9.5%, 9.2%, and 7.4% of all OTUs, respectively. On the other hand, in the RS, Enterobacteriaceae (20.6%), Bacillaceae (15.4%) and Sphingomonadaceae (11.7%) were the most OTU‐rich families. At the genus level, *Candidatus Portiera* and *Alicyclobacillus* were the most abundant in the VS, representing about 13.2% and 12.9%, respectively, followed by *Pseudonocardia* (10.2%) and *Sphingomonas* (9.8%). The relative abundance of other genera ranged between 0.4% and 5%. In the RS, *Sphingomonas* was the predominant genus, comprising 18.3% of total sequences. *Brevibacterium* (16.4%), *Pseudomonas* (16.3%), *Alicyclobacillus* (15.2%) and *Bacillus* (11.3%) were the other main genera detected in the samples.

**Figure 4:**
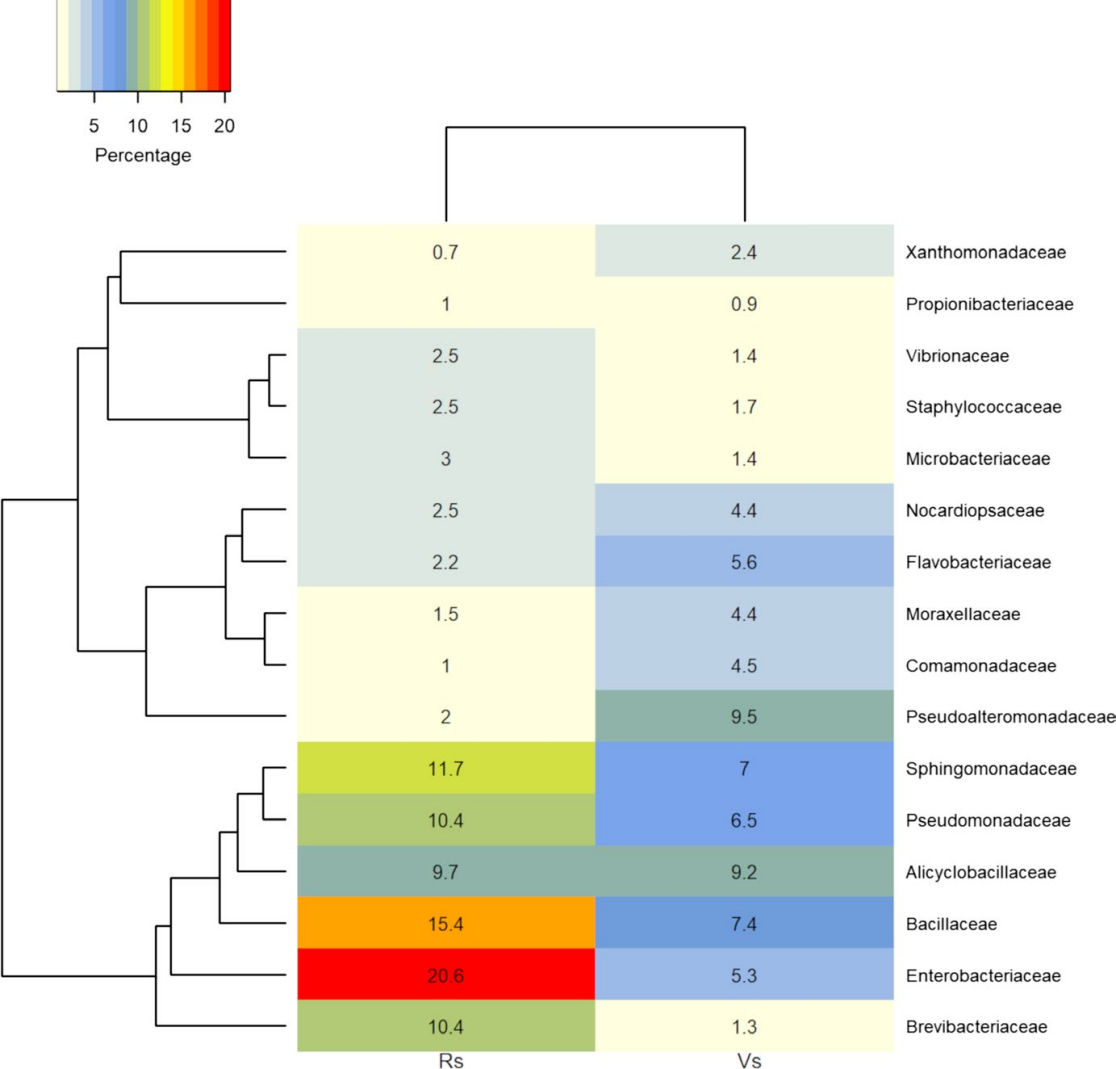
Heat map of the bacterial community composition of each stage based on Bray–Curtis distance. Taxonomic distribution of the core endophytic bacterial community at family level, based on an analysis of the first 16 most abundant families. Clustering of samples based on Bray–Curtis distance indices calculated by operational taxonomy units at a distance of 3%

Because the taxonomic assignment had a better resolution at the family level, the specific bacterial populations were statistically analyzed at this level. The heat map graph (Figure 4) was based on the 16 most abundant bacterial families. This analysis allowed us to find which taxonomic groups were most abundant in each developmental stage. The VS presented a larger abundance of families Pseudoalteromonadaceae, Alicyclobacillaceae, Bacillaceae, Sphingomonadaceae, and Pseudomonadaceae. In the RS, the families Enterobacteriaceae, Bacillaceae, Sphingomonadaceae, Brevibacteriaceae, and Pseudomonadaceae were more frequent.

**Figure A1:**
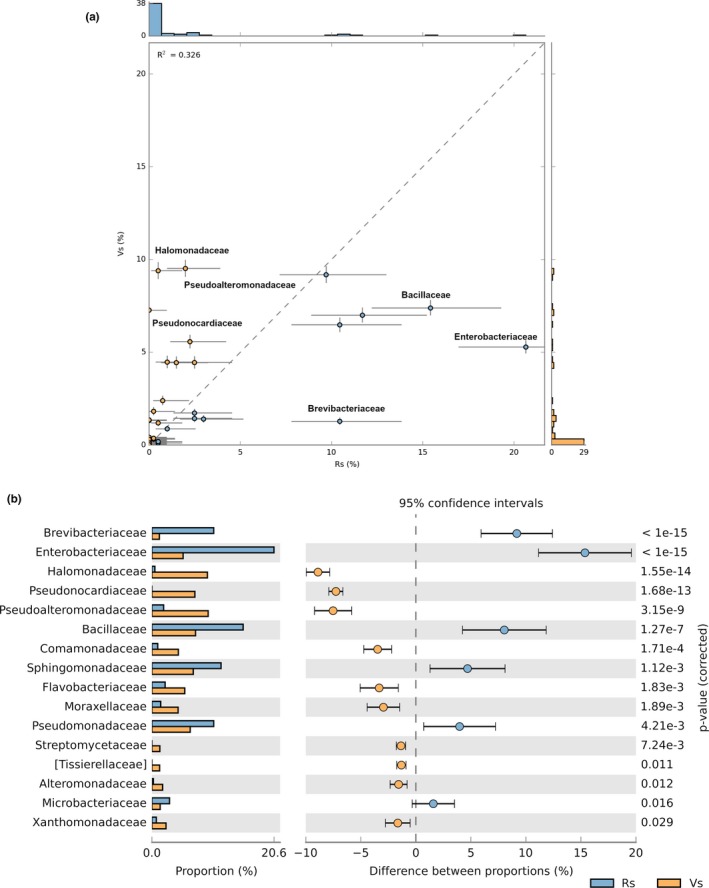
Comparison of taxonomic profiles between bacterial communities of vegetative and reproductive stages. (a) Scatter plot graph showing differences at the family level, (b) extended error bar percentage representation plot is showing differences in abundance between central bacterial populations at family level.

When leading bacterial families of each developmental stage were compared using the STAMP software (Appendix Figure A1), a significant overrepresentation of the Enterobacteriaceae, Brevibacteriaceae, and Bacillaceae was observed in the RS. Analysis of the VS showed a significant overrepresentation of Halomonadaceae, Pseudoalteromonadaceae, and Pseudonocardiaceae.

## 4 DISCUSSION

This study is a first effort toward the characterization of endophytic bacterial communities associated with passionflower (*P.  incarnata*), especially its leaves, analyzed by culture‐dependent and independent methods, comparing the two stages of plant development. The combination of both methods is highly recommended because it captures the microbial community structure and composition more precisely than when applying only one method, independently (Al‐Awadhi et al., 2013). Our results show the occurrence of specific endophytic populations at each developmental stages of this host (vegetative and reproductive). Endophytic groups that possibly boost plant growth were predominant at the VS, while groups associated with plant resistance and protection occurred more frequently at the RS.

The main disadvantage of culture‐based techniques is that they typically allow for the detection of no more than 0.1%–10% of true bacterial diversity within an ecosystem (Handelsman & Smalla, 2003; Pace, 1997), compared to the diversity obtained from culture‐independent techniques. However, in this study, the number of bacterial species (84 total) recovered by plating represented almost 20% of the overall number of OTUs (430 total) detected by Illumina‐based sequencing. There was an exclusive occurrence of some families within the cultivable diversity (Bradyrhizobiaceae, Micrococcaceae, Mycobacteriaceae, Erwiniaceae, Nocardiaceae), which emphasizes the importance of combining approaches (Thomas & Sekhar, 2017; Yashiro, Spear, & McManus, 2011). The exclusive occurrence of some bacterial taxa in cultivable diversity is not uncommon, since the culture‐independent approach may face limitations, such as the heterogeneous lysis of some bacterial species or low specificity of primers (Hill et al., 2000; Kennedy, Hall, Lynch, Moreno‐Hagelsieb, & Neufeld, 2014). On the other hand, the significant difference in the number of OTUs obtained by Illumina‐based sequencing compared with culturing may be explained by the inherent limitations of culture‐dependent methods and the undoubted capability of the next‐generation sequencing platform in producing large data sets (Tang, Ma, Li, & Li, 2016; Yang, Liu, & Ye, 2017). The diversification of culture media, expanding available nutrient sources, is considered a smart strategy to overcome the limitations of a culture‐dependent approach, because it favors the isolation of a broader range of bacterial populations. Despite most studies on isolation of leaf endophytic bacteria reporting low species richness (Gagne‐Bourgue et al., 2013; de Oliveira Costa, Queiroz, Borges, Moraes, & Araújo, 2012; Rhoden, Garcia, Santos e Silva, Azevedo, & Pamphile, 2015; Singh et al., 2017), our culture‐based approach allowed us to recover the highest number of species compared to the studies mentioned above. The species richness calculated from each culture medium was compared based on D_MG_. The highest species richness was obtained from the samples of the VS plated on the 869 medium. This culture medium comprises ingredients (glucose, yeast extract and tryptone) commonly used to recover nonfastidious microorganisms, which explains the observed dominance of easily cultivable bacterial species belonging to the families Bacillaceae and Pseudomonadaceae (Eevers et al., 2015). In the RS, the highest species richness was obtained on the Glycerol‐Asparagine medium, which contains glycerol as carbon source, asparagine as amino acid source, and various trace minerals; this combination of ingredients allowed for the isolation of a wide range of bacterial species belonging to the Sphingomonadaceae, Methylobacteriaceae, and Microbacteriaceae families (Huang et al., 2012; Li et al., 2005; Veyisoglu et al., 2013). Their growth rate may also have contributed to the predominance of these taxonomic groups in the culture media mentioned above.

Regarding the culture‐independent approach, rarefaction analyses from both samples suggested that the bacterial diversity in the RS was lower than that in the VS. Another possible bias affecting the culture‐independent analysis was the high percentage of sequences of plastid and mitochondrial origin yielded, though specific primers were used to avoid the amplification of plant organelle sequences (Chelius & Triplett, 2001). Similar results were found in a study on endophytic bacterial communities from banana shoot‐tip tissues (Yashiro et al., 2011). Additionally, 15% of the overall sequences from both the vegetative and RS corresponded to unassigned OTUs. A similar undetermined fraction was found in bacterial communities from the rhizosphere of amylaceous maize (Correa‐Galeote, Bedmar, Fernández‐González, Fernández‐López, & Arone, 2016), which indicates the occurrence of yet uncultured bacterial groups. However, as occurred in the study just cited, the unassigned sequences had no affected clustering (Dohrmann et al., 2013). The heat map analysis showed that the RS has more overrepresented bacterial groups than the VS. This differentially predominant grouping (seen in the heatmap graph) of several bacterial groups in the RS may constitute a defense against pathogen invasion that consequently would contribute to the plant’s health (Mendes, Raaijmakers, Hollander, Mendes, & Tsai, 2017). Alpha‐diversity indices indicate that the VS shows a vast superiority in species richness (Chao1 and Ace) over the RS, while values for Shannon and Simpson indices are very similar for both samples. The low endophytic richness values followed by an overrepresentation of specific microbial groups in the RS were also observed in the endophytic community from *Sequoia sempervirens* leaves, in which an association of lower species richness with higher leaf age was observed (Espinosa‐Garcia & Langenheim, 1990). Additionally, a study led by Andreolli, Lampis, Zapparoli, Angelini, and Vallini (2016) showed that species richness in an endophytic bacterial community associated with *Vitis vinifera* cv. Corvina is higher on 3‐year‐old grapevines than on 15‐year‐old ones. These changes in diversity may occur due to the loss of “passenger” endophytic populations within senescent leaves and, consequently, they may lead to the permanence and establishment of endophytes with critical ecological roles for the most advanced plant developmental stages. On the other hand, the decrease of nutrients in more advanced developmental stages may also make the host‐plant a less attractive niche for endophytic colonization, since in many conifers it was reported that mineral and sugar contents change as leaves age (Distelbarth, Kull, & Jeremias, 1984).

The study of host‐associated microbial community composition and structure may elucidate the ecological role that each microbial group plays within the phytobiome. Moreover, host development and health are dependent on the presence of an entire microbial community (Robinson, Bohannan, & Young, 2010). This study revealed that the genera *Bacillus* and *Pseudomonas* outnumber other cultivable bacteria in the host’s VS. The dominance of *Bacillus* isolates in the VS was also observed in a similar study where this genus made up to 90% of the entire endophytic bacteria in the early developmental stage of Ginseng (*Panax ginseng*) (Vendan, Yu, Lee, & Rhee, 2010). Similar results were reported on the abundance of *Bacillus* and *Pseudomonas* recovered from *Trichilia elegans* leaves (Rhoden et al., 2015). Besides being commonly characterized as endophytes (Govindasamy et al., 2010), *Bacillus* and *Pseudomonas* play a critical role in the promoting plant growth (Adesemoye, Obini, & Ugoji, 2008; Mercado‐Blanco & Bakker, 2007; Pérez, Collavino, Sansberro, Mroginski, & Galdeano, 2016). The genera *Sphingomonas*, *Curtobacterium*, and *Methylobacterium* together represented more than half of all bacteria isolated from the RS. The dominance of these three genera was also found in a study on cultivable endophytic bacteria associated with yerba mate (*Ilex paraguariensis*) (Araújo et al., 2002). Additionally, some previous studies showed that the occurrence of *Curtobacterium* and *Methylobacterium* has a particular influence on the acquisition of resistance to diseases caused by the phytopathogenic bacteria *Xylella fastidiosa* (Lacava, Araújo, Marcon, Maccheroni, & Azevedo, 2004; Sturz & Matheson, 1996) and *Erwinia caratovora* var. *atroseptica* (Ren et al., 2015), which means they may contribute to host‐plant health.

Taxonomic composition from the culture‐independent analysis was relatively similar to the cultivable diversity, mainly because the phyla Proteobacteria, Firmicutes, and Actinobacteria were the dominant groups in both analyses. Although Bacillaceae (third in abundance) was not the most abundant group in the VS, it kept its specific relevance in the community structure. The abundance of Bacillaceae in the VS of *P.  incarnata* was similar to a culture‐independent study showing that Bacillaceae was the fourth most abundant family of the entire leaf endophytic bacterial communities in the tillering stage (part of the VS) of rice cultivation (Lafi et al., 2016). The dominance of Alicyclobacillaceae in the VS is of particular interest due to the potential of extant members of this family to promote plant development and health (Suebphankoy, Sookanun, Na Chiangmai, Sawangsri, & Kanjanamaneesathian, 2013). For example, they can mitigate the adverse effects of heat and cold stress on plants (Xu et al., 2017). This finding might explain the relationship between the bacterial groups associated with promoting plant growth and the VS in *P.  incarnata*. In the structure of cultivable bacterial communities, Sphingomonadaceae was one of the most abundant families in the RS. This dominance was statistically confirmed by the analysis of taxonomic distribution between the two developmental stages conducted on STAMP.

Plants display specific physiological needs at each stage of development, which may be met by the occurrence of beneficial and critical microbial groups capable of boosting the host’s health. During the VS of the host‐plant, various metabolites are produced and mobilized for the growth of stems, branches, and leaves. Crucially, within the plant grows demand for nutrients such as nitrogen, phosphorus or iron, which are not always bioavailable (Crowley, 2006; Gupta, Panwar, Akhtar, & Jha, 2012; Hartmann, Schmid, Tuinen, & Berg, 2009). Therefore, the microbial groups that facilitate the intake of these nutrients could be selected by the plant and coevolve with it to supply for the physiological needs of the VS (Hartmann et al., 2009; Santoyo, Moreno‐Hagelsieb, Orozco‐Mosqueda Mdel, & Glick, 2016). This hypothesis was confirmed in this study, as the dominant taxonomic groups in the VS are commonly characterized as plant growth promoter microorganisms (Govindasamy et al., 2010; Lafi et al., 2016; Mapelli et al., 2013; Mercado‐Blanco & Bakker, 2007). On the other hand, there is a phenomenon in which plants gradually acquire resistance to pathogens during their life cycle; in the case of resistant plants, they increase with age their ability to control infections (Develey‐Rivière & Galiana, 2007). Thus, plants are generally more resistant in the most advanced developmental stages. The dynamic of the host‐associated bacterial communities may explain this phenomenon. During its development, the plant host may show a predisposition to be colonized by bacterial populations that participate in the defense against pathogens (Develey‐Rivière & Galiana, 2007; Sturz & Matheson, 1996). This might explain why predominant taxonomic groups in the RS are heavily related to bacterial groups that have previously shown influence on resistance to some infectious diseases (Araújo et al., 2002; Lacava et al., 2004; Pérez et al., 2016; Sturz & Matheson, 1996). Further studies are needed to assess host‐endosymbiont metabolomics at different developmental stages and determine whether the structure and composition of the endophytic bacterial communities could correlate with the plant phenological patterns.

## 5 CONCLUSIONS

This study revealed the existence of differentiated communities according to the developmental stage of the plant. Both the culture‐dependent and culture‐independent approaches showed that specific bacterial populations were exceptionally abundant for each developmental stage, which may be due to endophyte selection being driven by physiological changes (such as nutritional requirements or susceptibility to pathogens) occurring during the host development.

## CONFLICT OF INTERESTS

The authors declare that there is no conflict of interest.

## AUTHOR CONTRIBUTIONS

FF‐G, DA‐A supervision, funding acquisition and resources. MCG, LGCY, KJHM formal analysis. MCG, LGCY conceptualization, visualization, writing—original draft preparation and writing—review and editing.

## ETHICS STATEMENT

None required.

## Data Availability

All data are provided in the results section of this paper. The sequence data for the isolates are available at http://www.ncbi.nlm.nih.gov/genbank/ under accession numbers from MG778707 to MG778907. The sequence data from Illumina MiSeq are available at https://www.ncbi.nlm.nih.gov/bioproject/PRJNA430160.

## APPENDIX 1

**Table A1:** Cultivable bacterial diversity associated with *Passiflora incarnata* leaves

Plant developmental stages					
Vegetative			Reproductive		
The closest neighbor	No. isolates	Accession no.	The closest neighbor	No. isolates	Accession no.
*Bacillus*	70.7%		*Sphingomonas*	32.6%	
*B. aryabhattai*	10	EF114313	*S. yabuuchiae*	10	AB071955
*B. megaterium*	9	JJMH01000057	*S. sanguinis*	8	BCTY01000091
*B. tequilensis*	7	AYTO01000043	*S. zeae*	8	KP999966
*B. safensis*	4	ASJD01000027	*S. parapaucimobilis*	4	BBPI01000114
*B. siamensis*	3	AJVF01000043	*S. panni*	3	AJ575818
*B. altitudinis*	1	ASJC01000029	*S. pseudosanguinis*	2	AM412238
*B. wiedmannii*	1	LOBC01000053	*S. leidyi*	2	AJ227812
*B. cereus*	1	AE016877	*S. melonis*	2	KB900605
*B. velezensis*	1	AY603658	*S. molluscorum*	2	AB248285
*B. anthracis*	1	AE016879	*S. insulae*	1	EF363714
*B. thuringiensis*	1	ACNF01000156	*S. paucimobilis*	1	BBJS01000072
*B. nealsonii*	1	EU656111	*Shingomonas *sp.	4	AORY01000018
*B. gibsonii*	1	X76446	*Curtobacterium*	16%	
*Pseudomonas*	8.6%		*C. oceanosedimentum*	14	EF592577
*P. psychrotolerans*	4	FMWB01000061	*C. herbarum*	5	AJ310413
*P. cichorii*	1	FNIK01000055	*C. pusillum*	2	AJ784400
*Pantoea*	6.9%		*C. albidum*	1	AM042692
*P. stewartii*	1	JPKO01000033	*C. plantarum*	1	JN175348
*P. ananatis*	2	JMJJ01000010	*Methylobacterium*	13.8%	
*P. vagans*	1	EF688012	*M. radiotolerans*	6	CP001001
*Paenibacillus*	3.5%		*M. goesingense*	3	AY364020
*P. barcinonensis*	1	AJ716019	*M. rhodesianum*	2	AB175642
*P. xylanaxedens*	1	CLG_48537	*M. populi*	2	CP001029
*Rhodococcus*	3.5%		*M. komagatae*	2	AB252201
*R. erythropolis*	2	BCRM01000055	*M. phyllostachyos*	1	jgi.1071174
*Lysinibacillus*	1.7%		*M. oryzae*	1	CP003811
*L. fusiformis*	1	AB271743	*M. marchantiae*	1	FJ157976
*Xanthomonas*	1.7%		*M. fujisawaense*	1	AB175634
*X. sacchari*	1	Y10766	*M. aquaticum*	1	LABX01000161
*Leclercia*	1.7%		*Paracoccus*	5.5%	
*L. adecarboxylata*	1	BCNP01000062	*P. yeei*	4	JHWH01000002
*Enterobacter*	1.7%		*P. marcusii*	3	Y12703
*E. cloacae*	1	AXOM01000004	*P. aerius*	1	KX664462
			*Acinetobacter*	4.8%	
			*A. johnsonii*	3	APON01000005
			*A. junii*	1	APPX01000010
			*A. radioresistens*	1	BAGY01000082
			*Acinetobacter *sp.	2	NHRN01000069
			*Bacillus*	4.2%	
			*B. siamensis*	2	AJVF01000043
			*B. gibsonii*	1	X76446
			*B. megaterium*	1	JJMH01000057
			*B. niacini*	1	AB021194
			*B. siralis*	1	AF071856
			*Microbacterium*	4.2%	
			*M. enclense*	5	KQ758481
			*M. proteolyticum*	1	KM359785
			*Pseudomonas*	2.8%	
			*P. azotoformans*	2	MNPV01000020
			*P. coleopterorum*	1	KM888184
			*P. hunanensis*	1	JX545210
			*Streptomyces*	2.1%	
			*S. achromogenes subsp. rubradiris*	3	AB184561
			*Xanthomonas*	1.4%	
			*X. alfalfae subsp. citrumelonis*	2	JX986962
			*Moraxella*	1.4%	
			*M. osloensis*	2	CP014234
			*Klebsiella*	1.4%	
			*K. michiganensis*	2	JQ070300
			*Enterobacter*	1.4%	
			*E. xiangfangensis*	2	FYBF01000083
			*Arthrobacter*	0.7%	
			*A. enclensis*	1	JF421614
			*Bradyrhizobium*	0.7%	
			*B. daqingense*	1	jgi.1041378
			*Brevundimonas*	0.7%	
			*B. albigilva*	1	KC733808
			*Chryseobacterium*	0.7%	
			*C. hominis*	1	jgi.1096633
			*Enhydrobacter*	0.7%	
			*E. aerosaccus*	1	AJ550856
			*Kitasatospora*	0.7%	
			*K. arboriphila*	1	AY442267
			*Leifsonia*	0.7%	
			*L. shinshuensis*	1	DQ232614
			*Metalliresistens*	0.7%	
			*M. boonkerdii*	1	EU177512
			*Micrococcus*	0.7%	
			*M. yunnanensis*	1	FJ214355
			*Pantoea*	0.7%	
			*P. vagans*	1	EF688012
			*Paenibacillus*	0.7%	
			*P. naphthalenovorans*	1	AF353681
			*Mycobacterium*	0.7%	
			*M. phocaicum*	1	AY859682
